# Wood Odor Mapping on Arousal Axes: Exploring Correspondence with Physiological Indices of Stress Recovery

**DOI:** 10.3390/ijerph22111716

**Published:** 2025-11-13

**Authors:** Takashi Shima, Kei Maeda, Yuko Tsunetsugu

**Affiliations:** 1 Department of Biomaterial Sciences, Graduate School of Agricultural and Life Sciences, The University of Tokyo, 1-1-1, Yayoi, Bunkyo-ku 113-8657, Japan; maeda_kei780@ffpri.go.jp; 2 Kajima Technical Research Institute, Kajima Corporation, 2-19-1, Tobitakyu, Chofu-shi 182-0036, Japan; 3 Forestry and Forest Products Research Institute, 1 Matsunosato, Tsukuba 305-8687, Japan

**Keywords:** wood odor, arousal level, stress recovery, physiological indices, indoor environment

## Abstract

This study mapped a wide range of naturally derived odors, including those derived from wood, on the two-dimensional axes of tense arousal (TA) and energetic arousal (EA), and examined whether quadrant differences influenced recovery following stress. In the context of Attention Restoration Theory and biophilic design, the study provided preliminary evidence that olfactory stimuli can be treated as a designable element in a functional and reproducible manner. In Experiment 1, wood flours, wood essential oils, and non-wood oils were mapped based on subjective ratings conducted under identical conditions, and differences in their TA–EA positions were revealed. Ratings of “naturalness” were associated with lower EA, suggesting that quadrant mapping can capture meaningful dimensions of odor perception. In Experiment 2, Hinoki and camphor were selected as contrasting stimuli. Hinoki facilitated initial recovery of autonomic nervous system activity, as shown by lower heart rate compared with no odor, whereas camphor showed no effect. These findings demonstrate that TA–EA quadrant mapping provides a practical framework for olfactory design in indoor environments.

## 1. Introduction

In contemporary society, the diversification of working styles and the increasing complexity of interpersonal relationships have subjected individuals to considerable daily stress [1]. This issue has become evident in Japan as well; a 2024 survey reported that more than 80% of people experience stress in office environments where they spend a large portion of their time [2]. To maintain a healthier lifestyle, it is crucial to have effective means of recovering from stress.

Traditional approaches to stress management include personnel- and system-oriented strategies such as task adjustment and the improvement of interpersonal relationships. In addition, growing attention has been paid to the potential of architectural spaces to promote recovery from mental fatigue through sensory stimulation and environmental features. This perspective is grounded in the Attention Restoration Theory (ART), proposed by Kaplan [3], which identifies four environmental properties—“fascination,” “being away,” “extent,” and “compatibility”—as facilitators of recovery from cognitive fatigue. Based on this theory, numerous studies have demonstrated the effectiveness of incorporating natural environments that embody these properties [4].

Against this theoretical background, the design philosophy of biophilic design has gained traction in recent years [5]. Rooted in the biophilia hypothesis—that humans have an inherent tendency to seek connection with nature—biophilic design incorporates elements such as vegetation, water features, natural light, external views, and natural materials to enhance comfort, creativity, and health for building occupants.

Among these strategies, particular attention has been directed toward the use of wooden materials in architectural spaces. Beyond their recognition as carbon-neutral materials—helping reduce CO_2_ emissions during production and serving as carbon sinks in the context of decarbonization efforts [6]—wooden materials also embody the restorative features highlighted in ART. As such, they are expected to contribute to improved occupant comfort, and a growing body of research has investigated these possibilities [7,8,9,10,11].

From this perspective, wood represents a tangible form of “nature within indoor environments” and uniquely provides a multi-sensory experience encompassing vision, touch, and olfaction. The present study focuses specifically on the olfactory dimension. In indoor applications, olfactory stimulation can be implemented through two main routes: (i) natural emission from wood itself (e.g., interior materials, furniture, wood flour) and (ii) presentation of extracted products (essential oils). As these routes differ in chemical composition and temporal release profiles, even scents collectively perceived as “woody” may exert distinct psychological and physiological effects.

A review of findings on wood-derived odors suggests that outcomes vary depending on species (e.g., Sugi [12]; Hinoki and Meniki [13]; Camphor [14]; and members of the *Cupressaceae* family [15]), odor components (e.g., α-pinene [16]), presentation mode (e.g., emission from interior materials [17,18] vs. essential oils [13,14]), and contextual conditions (e.g., rest [16] vs. task engagement [19]). Overall, autonomic nervous system (ANS) responses tend to shift toward relaxation [16,17], while central nervous system (CNS) responses show both relaxation [16] and activation [14,20,21]. This apparent “divergence” suggests that odors may simultaneously influence two independent dimensions: tension and vitality.

This duality aligns with broader conceptualizations of physiological arousal as multidimensional. For example, Thayer [22] emphasized the importance of distinguishing between tense arousal (TA) and energetic arousal (EA) in understanding mood. Similarly, Matthews et al. [23] proposed a two-dimensional model of arousal levels and developed the University of Wales Institute of Science and Technology (UWIST) Mood Adjective Checklist, which separates arousal into two axes: EA, representing CNS-related activation, and TA, representing ANS-related activation. This framework is both theoretically robust and practically interpretable; for instance, in office environments, the desired state is often to “reduce tension without excessively diminishing vitality.” However, research investigating physiological and psychological responses to wood has not yet employed such models.

The present study therefore assumes that the inconsistencies observed across prior studies are attributable to differences in stimuli and contextual conditions that shift the coordinate positions along the TA and EA axes. To examine this, we adopt a two-dimensional arousal framework based on mood adjectives, allowing tension and vitality to be expressed as coordinates on the same plane. This framework enables explicit comparisons of functional differences (e.g., “reducing tension while maintaining vitality”) according to purpose and context.

To ensure the validity of this coordinate-based representation, the breadth and composition of stimuli are essential. Accordingly, we selected multiple wood species to reflect the diversity of wood characteristics. For each species, both wood flour (representing mild and complex emissions closer to natural conditions) and essential oils of the same species were included, alongside single-component essential oils extracted from key constituents. In addition, essential oils commonly used in aromatherapy—including non-wood species—were incorporated as reference stimuli. Evaluating all stimuli under unified presentation conditions enables standardized mapping of differences across presentation modes (wood flour vs. essential oil), species, and wood-derived vs. other essential oils.

Experiment 1 (standardized mapping) was designed to present wood flours, essential oils of the same species, and aromatherapy-related essential oils under identical conditions, and to determine their positions along the TA/EA axes (including reliability assessment). The aim was to standardize the relative positions of wood flours and essential oils, interspecies differences, and the positions of wood-derived vs. other essential oils on a common coordinate system.

Based on the results of Experiment 1, Experiment 2 (preliminary quadrant validation) focused on two wood-derived essential oils that were located in different quadrants of the TA/EA map. This experiment examined whether differences in recovery indices (autonomic responses, brain activity, and subjective evaluations) emerged between groups during rest. Only essential oils were used, and presentation conditions such as concentration and duration were standardized. Importantly, the TA/EA map itself was not assumed to be a predictive model; rather, Experiment 2 sought to preliminarily verify whether quadrant differences correspond to recovery outcomes.

In summary, the purpose of this study was to standardize wood-derived and other essential oils within a two-dimensional arousal framework and to verify whether differences in quadrants lead to significant variations in recovery during rest. The study tested the following hypotheses:

**H1:**  *Differences in odor type, chemical composition, and presentation mode (wood flour vs. essential oil) systematically influence the TA/EA coordinates of the stimuli.*

**H2:**  *TA/EA coordinates obtained in Experiment 1 correspond to the directionality of recovery indices in Experiment 2. Specifically, TA is expected to reflect tendencies in ANS responses, whereas EA is expected to correspond to CNS and vitality-related indices.*

The contributions of this study are twofold. First, it redefined odor effects in terms of the dual dimensions of tension and vitality, presenting a standardized TA/EA mapping followed by quadrant validation. Second, it provided preliminary evidence that differences in profiles derived from mood adjectives correspond with changes in recovery indices during rest, thereby offering insights to guide design decisions regarding the use of extracted scents versus actual wooden materials.

Beyond its implications for environmental design, understanding how wood-derived odors facilitate psychological recovery and stress regulation contributes directly to behavioral health promotion. These findings may inform evidence-based interventions and spatial design strategies aimed at reducing stress, enhancing relaxation, and promoting overall mental well-being in everyday and occupational environments.

## 2. Materials and Methods Introduction

### 2.1. Study Design

This study comprised two experiments. In Experiment 1, we obtained subjective evaluations of a wide range of woody and non-woody olfactory stimuli and mapped each stimulus on two axes—TA and EA. In Experiment 2, based on the results of Experiment 1, we selected scents from two wood species positioned in different quadrants and introduced them during rest following a stress-inducing task to examine recovery in physiological and psychological indices. Both experiments were approved by the ethics committee of The University of Tokyo.

### 2.2. Experiment 1

#### 2.2.1. Overview

The experiment was conducted in an indoor booth specifically prepared for olfactory testing. To minimize environmental influences, the booth was enclosed on three sides by white boards, creating a controlled setting for stimulus presentation. Participants were 12 adults in their 20s (9 men, 3 women; 22.0 ± 1.0 years). None of the participants had professional experience in forestry, wood science, or related industries. They were recruited from a general university population and were not informed of the specific purpose or hypotheses of the study to minimize expectancy bias. All participants reported normal olfactory and visual acuity and no known allergies to essential oils. Each participant completed the entire session in one day, lasting approximately 40 min including questionnaires. They sequentially smelled 19 odor stimuli and provided subjective ratings for each. The experiment was conducted in a temperature-controlled booth maintained at 20 °C and approximately 50% relative humidity. This temperature is consistent with previous olfactory studies that employed similar ambient conditions to ensure stable volatilization and participant comfort [18]. Before each session, participants were verbally asked whether they felt cold, and all reported that the temperature was comfortable.

**Figure 1:**
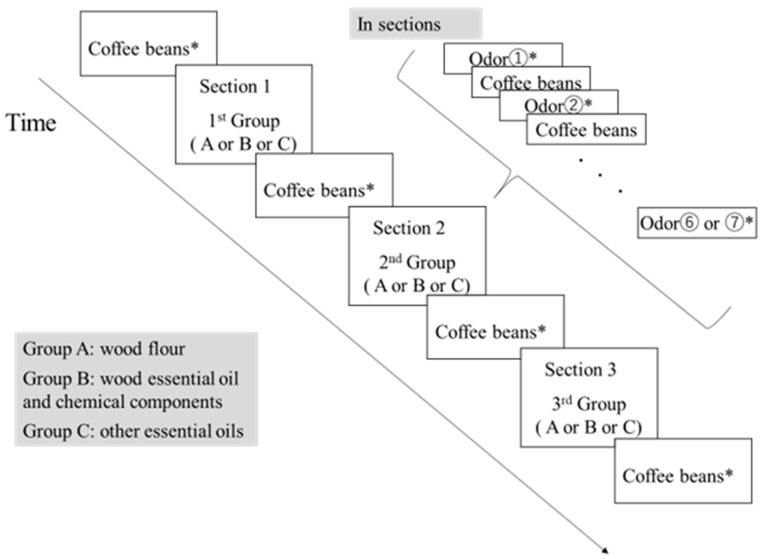
Experimental procedure (Experiment 1). *: Time allotted to respond to the subjective evaluation questionnaire.

Stimuli comprised three groups—six wood flours, seven wood essential oils and their odor components, and six non-wood essential oils—for a total of 19 stimuli. The session was divided into three sections; in each section, one stimulus group was presented and subjective ratings were obtained for every stimulus (Figure 1). To minimize carryover effects, the odor of coffee beans were presented between stimuli following prior research [24]. Subjective ratings for coffee beans were also obtained at the beginning and end of the session and at each section switch. The order of the three groups and the order of stimuli within each section were randomized for each participant (Figure 1).

**Figure 2:**
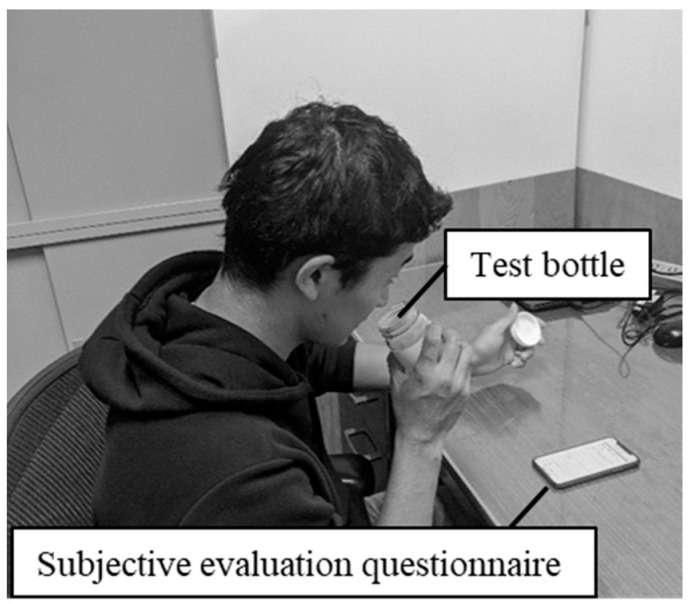
Introduction of olfactory stimulation.

The odors were presented using the odor-bottle method [25] with 200 mL glass bottles for all stimuli (Figure 2). The bottles were wrapped in opaque white paper so that participants could not see the contents. This visual masking prevented cognitive bias from color or texture cues and ensured that evaluations were based solely on olfactory perception. After smelling the bottle of coffee beans, participants exhaled nasally three times and then slowly smelled the test bottle handed to them by the experimenter without viewing its contents. Odor intensity was rated within the first three breaths; participants were permitted to re-smell during questionnaire responses if needed.

#### 2.2.2. Types of Stimuli

Wood flours were prepared from small chips of Hinoki (*Chamaecyparis obtusa*), Sugi (*Cryptomeria japonica*), Hiba (*Thujopsis dolabrata*), camphor (*Cinnamomum camphora*), and Japanese white pine (*Pinus parviflora*). Materials were milled with a Wiley mill to ≤2 mm and, in principle, 0.5 g was placed in each test bottle. Wood flours were prepared from sapwood of each species. Prior to milling, all samples were air-dried at room temperature for two weeks, then stored in sealed polyethylene bags under dark, dry conditions until use. No heating, freezing, or chemical treatment was applied. Species selection was based on prior reports of psychological and physiological effects of wood and wood essential-oil odors [8]: we included Hinoki and Sugi, for which numerous findings exist, and tested both wood flour and commercially available essential oils. In addition, we used wood flours of Hiba (the next most frequently studied), Japanese white pine (for which increased alpha-wave incidence has been reported [26]), and camphor (reported to suppress stress-hormone secretion during calculation tasks [27]). Dosages were determined by a pilot test using the six-point odor-intensity scale [28] to fall between “just detectable” and “strong” for all species. Because Hinoki wood flour was judged markedly stronger than Hinoki essential oil, we also included a 0.15 g Hinoki wood-flour condition.

We further added odor components known to constitute wood odors when commercially available as reagents. Wood essential oils were Hinoki and Sugi (both from TREE OF LIFE Co., Ltd., Tokyo, Japan). Odor components were α-pinene, limonene, 1,8-cineole (eucalyptol), linalool, and α-terpineol (all from FUJIFILM Wako Pure Chemical Corporation). Based on pilot tests, volumes were 30 µL for Sugi and Hinoki essential oils, 20 µL for each component, and 0.1 g for α-terpineol (solid at room temperature). Except for α-terpineol, liquids were applied to scent strips and enclosed in the test bottles.

To clarify the relative positions of wood odors on the map, we also included commonly marketed aromatherapy essential oils frequently studied in research as comparators, and evaluated them in the same manner. Non-wood essential oils were bergamot, lavender, lemon, peppermint, ylang-ylang, and grapefruit (all from Tree of Life Co., Ltd.). These essential oils were selected because they are widely used in aromatherapy. Thus, the purpose of this study was not to emphasize wood scents as inherently positive, but to position them among widely familiar natural aromas for standardized comparison. Based on pilot tests, 20 µL of each was applied to scent strips for presentation.

#### 2.2.3. Subjective Evaluations

Subjective measures comprised: (i) the 11-point odor-intensity scale; (ii) the Japanese version of the UWIST Mood Adjective Checklist (JUMACL) [29], which evaluates TA and EA; and (iii) nine impression ratings on seven-point scales. Although the odor-intensity scale is nominally six-point, we used 11 points by including midpoints between adjacent categories. The nine impression items were “mild–stinging,” “clearly–misty,” “relaxed–fidgety,” “refreshed–depressed,” “calm–irritated,” “good–bad,” “like–dislike,” “natural–artificial,” and “familiar–unfamiliar.” The first five items represent factors (softness, clarity, relaxation, elation, stress) reported by Higuchi et al. [30]; the last four were selected with reference to a similar experiment evaluating wood scents [21].

#### 2.2.4. Statistical Analysis

Associations among subjective indices across all odors were assessed using Pearson product–moment correlation coefficients. Because the normality assumption was not satisfied for some variables (Shapiro–Wilk test, *p* < 0.05), Spearman’s rank correlation was also calculated to confirm the robustness of the relationships. Both analyses showed consistent tendencies, and no major outliers were detected. For impression items, we conducted principal component analysis (PCA) and reported explained variance for each component and correlations of component scores with EA and TA. To categorize patterns of EA and TA scores, we performed hierarchical cluster analysis (squared Euclidean distance; Ward’s method). Differences between clusters were tested with the Mann–Whitney U test and adjusted by the Bonferroni method.

Within-species differences in subjective ratings were compared across presentation modes. For Hinoki (wood flour 0.5 g, wood flour 0.15 g, essential oil), we applied the Friedman test to each subjective item; when significant, we used the Wilcoxon signed-rank test with Bonferroni correction. For Sugi (wood flour 0.5 g and essential oil), the essential-oil condition was added mid-study; therefore, seven participants contributed to that condition. Condition comparisons for Sugi used the Mann–Whitney U test.

Potential effects of presentation order and olfactory fatigue were examined using repeated ratings of coffee beans throughout the session; differences across time points were tested by the Friedman test. The alpha level was set at 5% for all analyses. The threshold *p* < 0.10 was treated as exploratory significance to detect potential tendencies under the small-sample pilot design, consistent with prior psychophysiological studies employing similar sample sizes (e.g., [19]). Analyses were conducted with SPSS Statistics ver. 25.

### 2.3. Experiment 2

#### 2.3.1. Overview

The experiment was conducted in a booth installed in a classroom at the Faculty of Agriculture, The University of Tokyo, from 30 November to 18 December 2020. Participants were 15 adults in their 20 s (12 men, 3 women; 22.1 ± 1.2 years). None of the participants had professional experience in forestry, wood science, or related industries. All participants reported normal olfactory and visual acuity and no known allergies to essential oils. Each participant completed all procedures in a single day.

**Figure 3:**
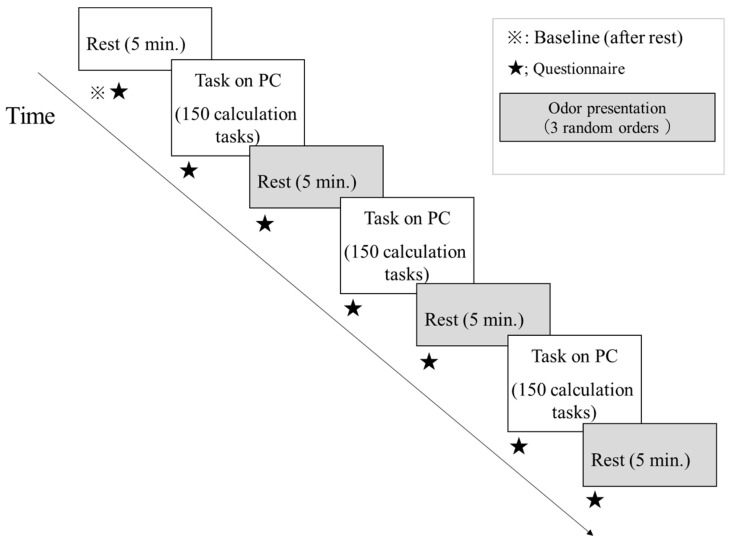
Experimental procedure (Experiment 2). During the three olfactory stimulus introduction phases, three conditions (no odor, Hinoki essential oil introduction, and camphor essential oil introduction) were implemented in random order. PC = personal computer used to present for the calculation task.

After receiving instructions, participants entered a booth maintained at 20 °C and approximately 50% relative humidity, following the same environmental setting as in Experiment 1 to ensure comparability between experiments and participant comfort. Participants then donned a commercially available activated-carbon mask (BMC Co., Ltd., Tokyo, Japan) and the physiological sensors described below, and completed baseline questionnaires. Participants were verbally asked whether they felt cold before each session; all confirmed that the temperature was comfortable. No participants reported thermal discomfort during the sessions. A 5 min resting period was recorded as baseline. Participants then performed a calculation task comprising 150 randomized problems (two-digit × one-digit multiplication or two-digit + two-digit addition). The calculation task was presented on a personal computer, and participants entered their responses directly on the screen. Numerical values were created using Microsoft Excel 2019 and randomized individually for each participant. The mean completion time was 9.7 ± 2.0 min, and the mean accuracy rate was 91.2 ± 11.0%. Accuracy scores were used solely to confirm task engagement and were not included in subsequent statistical analyses. The calculation task followed the procedure described by Shima et al. [21], which was originally designed to induce mild cognitive stress in a controlled laboratory setting. However, our previous study (Shima et al., 2020 [21]) revealed that this task was relatively easy for participants and did not produce sufficient stress responses. Therefore, in the present experiment, the difficulty level and number of problems were slightly increased to enhance the stress-inducing effect while maintaining ethical and safety considerations. Immediately after task completion, the mask was replaced to introduce the odor condition, followed by a 5 min rest. This calculation-task/rest sequence was repeated three times (Figure 3). This intervention paradigm—repeated task–rest cycles to test effects of olfactory stimuli—has been used in prior studies [19,31], and our protocol was designed with reference to them. Subjective ratings were obtained immediately before each rest (just after odor introduction) and immediately after each 5 min rest.

#### 2.3.2. Odor Presentation

Essential oil was introduced during each rest by applying drops onto the mask. Conditions were: Hinoki 20 µL, camphor 20 µL, and no-odor. Essential oils were dispensed using a 10 µL micropipette onto the outer surface at the center of the mask (nose region). Masks were prepared immediately before each rest phase to prevent volatilization loss and were worn immediately after application. The order of the three conditions was randomized within participants. Volumes were determined by pilot testing based on the six-point odor-intensity scale to fall between “just detectable” and “strong.” Selection of scents followed the policy from Experiment 1 to use two wood species located in different TA/EA classification.

#### 2.3.3. Measurements

Physiological measures included skin conductance level (SCL), heart rate (HR), and heart rate variability (HRV) as ANS indices, and oxygenated hemoglobin concentration (O_2_Hb) as a CNS index. All are biomarkers reported to change with exposure to wooden environments. SCL was recorded using the AP108 physiological recorder with AP-U030 electrodermal unit (Miyuki Giken Co., Ltd., Tokyo, Japan) at 500 Hz using a constant-current method. HR and HRV were obtained from R–R intervals with the WHS-1 heart monitor (Union Tool Co., Ltd., Tokyo, Japan), and power in the low-frequency (0.04–0.15 Hz; LF) and high-frequency (0.15–0.40 Hz; HF) bands and the LF/HF ratio were computed by the maximum-entropy method. HF predominantly reflects parasympathetic activity, and LF/HF predominantly reflects sympathetic activity. O_2_Hb was measured by near-infrared spectroscopy (NIRS) using the NIRO-300 cerebral oximeter (Hamamatsu Photonics), with sensors attached to the left and right forehead and sampled at 1 Hz. Psychological measures were TA and EA assessed with JUMACL.

#### 2.3.4. Statistical Analysis

For physiological indices, we analyzed data from the last 1 min of the calculation task through the end of the subsequent 5 min rest, computing 1-min means. For each time point, baseline (resting) values were subtracted to create difference series for analysis. The main analysis used repeated-measures ANOVA with within-subject factors of Condition (no odor, Hinoki, camphor) and Time (1 min bins during rest). When a significant Time effect was observed, post hoc pairwise comparisons between time points were performed on the series averaged across the three conditions with Bonferroni correction. In addition, even when the Condition × Time interaction was not significant, we inspected p-values for all pairwise comparisons among the three conditions at each time bin to assess potential differences in the immediacy of physiological responses. For O_2_Hb, the bilateral mean (left–right average) was analyzed, and three participants with device failure were excluded (analysis sample N = 12).

For psychological indices (TA and EA from JUMACL), we applied the Friedman test to factors of Time (pre- vs. post-rest; baseline excluded) and Condition (no odor, Hinoki, camphor). When significant, Wilcoxon signed-rank tests with Bonferroni correction were conducted.

For all statistical tests, the alpha level was 5%, and *p* < 0.10 was treated as an exploratory trend. Analyses were conducted with SPSS Statistics ver. 25.

## 3. Results

### 3.1. Mapping Odor Responses on Arousal Axes (Experiment 1)

**Figure 4:**
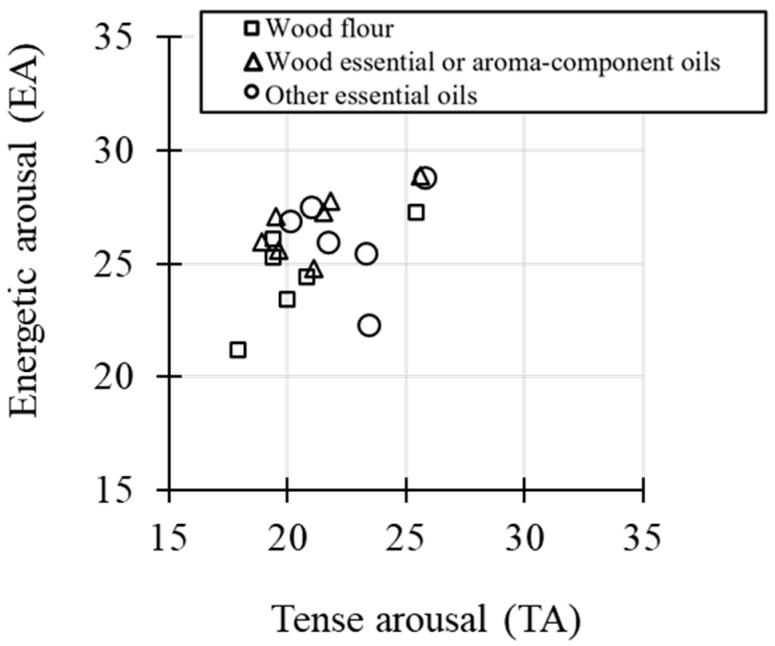
Scatter plot of energetic arousal versus tense arousal (N = 19).

Across all 19 odor stimuli, EA and TA scores from JUMACL showed a significant positive correlation (r = 0.49, *p* = 0.03; Figure 4). The neutral point of the scale was (25, 25). Among wood-flour stimuli, Hinoki and camphor were positioned with lower TA and higher EA than the neutral point, whereas Sugi, Japanese white pine, and Hiba were located in the low-TA and low-EA quadrant. Sugi exhibited the lowest EA and TA among all stimuli. Wood essential oils and single odor components generally distributed in the low-TA, high-EA quadrant (Figure 4).

PCA of odor intensity and impression ratings extracted two components, with a cumulative explained variance of 89.6% (Table 1). The first component score was strongly negatively correlated with TA (r = −0.92, *p* < 0.001) and also negatively correlated with EA (r = −0.57, *p* = 0.01). The second component score was positively correlated with EA (r = 0.68, *p* = 0.001). Table 2 summarizes the intercorrelations among subjective measures.

**Figure 5:**
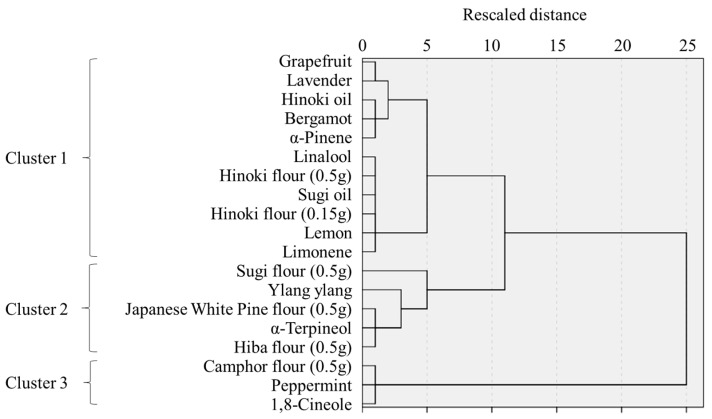
Dendrogram of odor types by hierarchical clustering.

**Figure 6:**
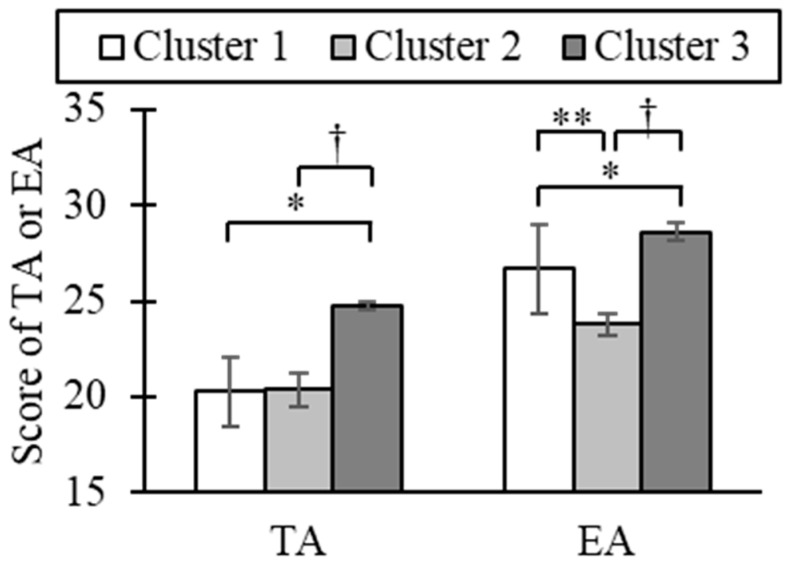
TA and EA scores of different odor clusters (Cluster 1: N = 3, Cluster 2: N = 5, Cluster 3: N = 11, mean ± SE, **: *p* < 0.01, *: *p* < 0.05, †: *p* < 0.1, Mann–Whitney U tests with the Bonferroni correction).

Hierarchical cluster analysis based on EA and TA scores classified stimuli into three clusters (Figure 5). Differences in TA among clusters showed a significant difference between clusters 1 and 3 (*p* = 0.03) and a marginal difference between clusters 1 and 2 (*p* = 0.07). For EA, simple main effects revealed significant or marginal differences across all cluster pairs (1 vs. 2: *p* = 0.006; 1 vs. 3: *p* = 0.03; 2 vs. 3: *p* = 0.08). Descriptively, cluster 1 was characterized by low TA and moderate EA, cluster 2 by low TA and low EA, and cluster 3 by high TA and high EA (Figure 6).

### 3.2. Within-Species Comparisons (Experiment 1)

**Figure 7:**
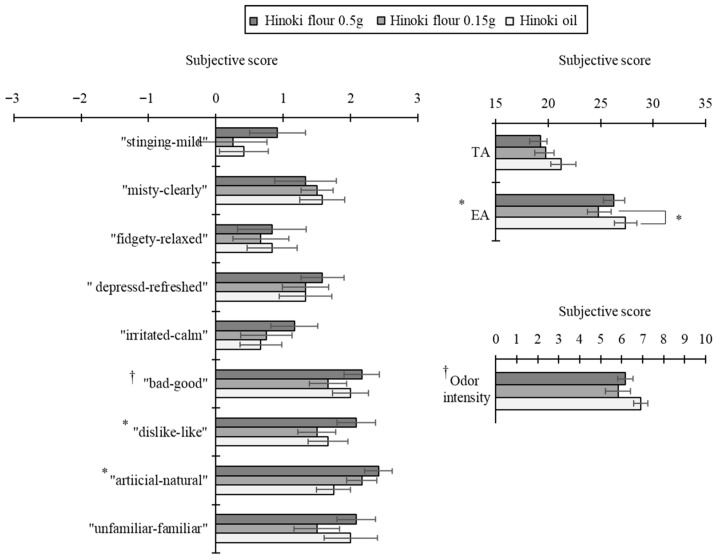
Subjective impression scores of the Hinoki flour and essential oil odors (N = 12, mean ± SE, *: *p* < 0.05, †: *p* < 0.1, Friedman test/Wilcoxon signed-rank tests with the Bonferroni correction).

**Figure 8:**
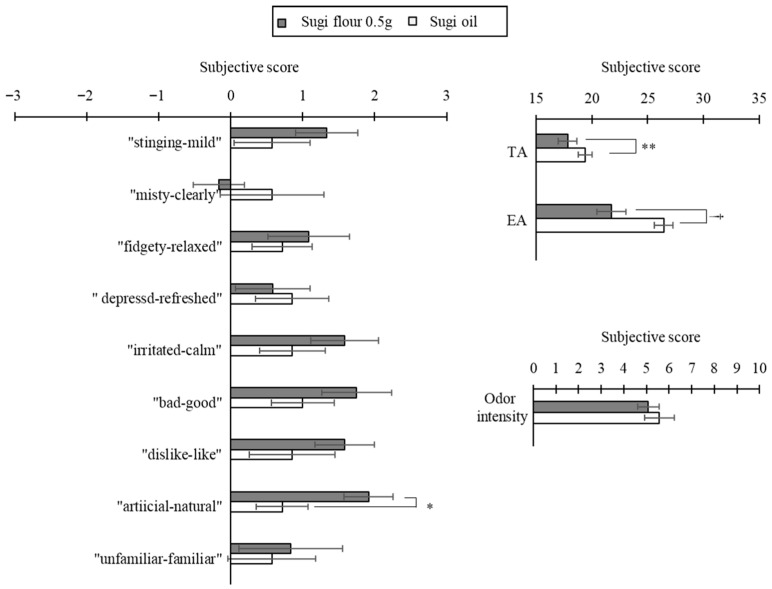
Subjective impression scores of the Hinoki flour and essential oil odors (Sugi flour: N = 12, Sugi oil: N = 7, mean ± SE, **: *p* < 0.01, *: *p* < 0.05, †: *p* < 0.1, Mann–Whitney U tests).

Within-species comparisons for Hinoki (wood flour 0.5 g, wood flour 0.15 g, essential oil) revealed significant or marginal differences in five subjective ratings: “good–bad” (*p* = 0.08), “like–dislike” (*p* = 0.03), “natural–artificial” (*p* = 0.05), odor intensity (*p* = 0.09), and TA (*p* = 0.01). Post hoc tests showed that compared to 0.15 g of wood flour, essential oil yielded significantly higher EA scores (*p* = 0.04; Figure 7). For Sugi, 0.5 g of wood flour was rated significantly more natural than essential oil on the “natural–artificial” scale (*p* = 0.03), and it produced significantly higher TA (*p* = 0.003) and a marginally higher EA (*p* = 0.08; Figure 8).

Analyses of olfactory fatigue and order effects showed that odor intensity ratings for coffee beans did not differ across four time points, nor were there differences in impression ratings, EA, or TA. No order effects were detected across all stimuli for any subjective measures.

### 3.3. Physiological Responses During Rest Periods (Experiment 2)

**Figure 9:**
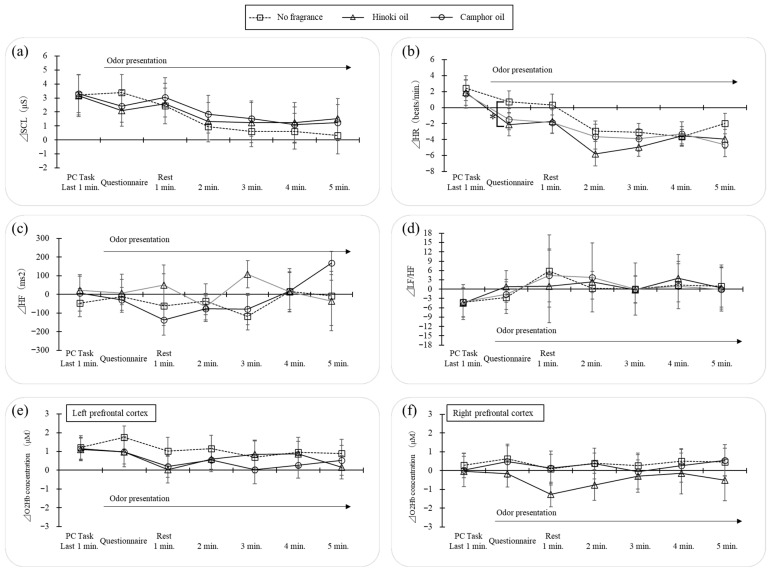
Time course of physiological indices during the post-task rest period (mean ± SE *: *p* < 0.05, *t*-test with the Bonferroni correction) (**a**) Skin conductance level (SCL), (**b**) heart rate (HR), (**c**) high-frequency power of heart rate variability (HF, log-transformed), (**d**) LF/HF ratio (log-transformed), (**e**) oxygenated hemoglobin concentration (O_2_Hb) in the left prefrontal cortex, and (**f**) O_2_Hb in the right prefrontal cortex. All values are expressed as differences from the baseline (resting) level, averaged for each minute from the last minute of the calculation task to the end of the 5-min rest. The analysis sample was N = 15 for all indices except O_2_Hb. For O_2_Hb, the bilateral mean was used, and three participants with device failure were excluded (analysis sample N = 12).

SCL, analyzed as 1-min means from the final minute of the calculation task to the end of rest (all values expressed as differences from baseline), showed a significant main effect of Time (*p* = 0.03), but no significant main effect of Condition or Condition × Time interaction (Figure 9a). For HR, a significant main effect of Time was also observed (*p* = 0.001), with no significant Condition effect or interaction (Figure 9b). In predefined pairwise comparisons at the questionnaire time point, Hinoki differed significantly from no odor (*p* = 0.04). Differences between Hinoki and camphor and between camphor and no odor were not significant after correction. No other between-condition differences at other time points were observed. For HF, LF/HF ratio, and O_2_Hb in the prefrontal cortex measured by NIRS, no significant main effects or interactions were detected (Figure 9c–f).

### 3.4. Subjective Arousal (Experiment 2)

**Figure 10:**
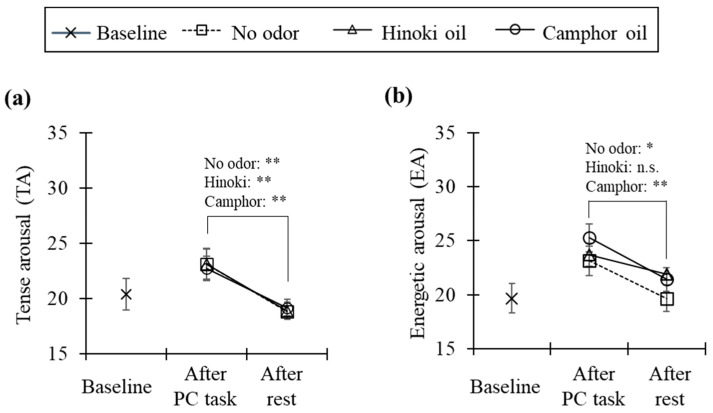
(**a**) Scores of tense arousal before and after rest; (**b**) Scores of energetic arousal before and after rest (mean ± SE, N = 15, **: *p* < 0.01, *: *p* < 0.05, n.s.: not significant, Wilcoxon signed-rank tests with the Bonferroni correction).

TA and EA scores from JUMACL (excluding baseline) across six conditions (pre- vs. post-rest × three odor conditions), together with baseline scores, are shown in Figure 10. For TA, scores significantly decreased after rest compared to before rest under all odor conditions (no odor: *p* < 0.002, Hinoki: *p* < 0.009, camphor: *p* < 0.007); however, no differences among species were observed at either pre- or post-rest (Figure 10a). For EA, scores significantly decreased after rest compared to before rest in the no odor (*p* < 0.01) and camphor conditions (*p* < 0.001), whereas Hinoki showed no significant decline. Nevertheless, no interspecies differences were observed at either pre- or post-rest (Figure 10b).

## 4. Discussion

This study mapped olfactory stimuli on the two-dimensional axes of JUMACL (TA and EA) and examined the relationship between their emotional profiles and recovery processes following stress. In Experiment 1, a wide range of wood-derived and non-wood odors were evaluated under uniform conditions and characterized by their positions on the quadrants. In Experiment 2, two stimuli classified into different clusters in Experiment 1 (Hinoki and camphor) were selected, and their effects on recovery during post-task rest were investigated in terms of both physiological and psychological responses.

According to Thayer’s two-dimensional model [22], combining TA and EA scores allows classification of affective states into four quadrants: high TA and high EA correspond to “tense energy,” low TA and high EA to “calm energy,” low TA and low EA to “calm tiredness,” and high TA and low EA to “tense tiredness.” The stimuli tested in this study were mainly located in the first two quadrants, with none classified as high TA/low EA. Experiment 1 revealed a positive correlation between EA and TA across all stimuli, indicating that the olfactory stimuli can simultaneously shift ANS-related tension and CNS-related vitality in the same direction. In a prior study [29], EA and TA were negatively correlated in pre-task assessments, suggesting that the tendency observed here may be unique to evaluations of olfactory stimuli. Species-specific analysis showed that Sugi wood flour was characterized by low EA and low TA, consistent with previous reports of sedative effects [32]. Camphor wood flour was positioned near tense energy, reflecting simultaneous increases in both tension and vitality, whereas Hinoki wood flour was closer to calm energy, indicating reduced tension while maintaining vitality.

Furthermore, Hinoki and Sugi wood flours were rated higher on positive impressions such as “mild,” “good,” and “like,” which corresponded to lower EA whereas essential oils tended to elevate EA slightly, suggesting functional differentiation depending on presentation form. PCA of impression ratings confirmed that the adjectives divided mainly into two groups, corresponding to TA and EA, validating the appropriateness of the arousal framework used in this study. Both Hinoki and Sugi wood flours were judged as more “natural” than their essential oils, and this naturalness was associated with lower EA, consistent with prior findings that perception of natural elements enhances relaxation [3]. Cluster analysis identified three groups, with stimuli containing 1,8-cineole (e.g., camphor wood flour and peppermint [33,34]) concentrated in the high-TA, high-EA group, suggesting a possible contribution of this compound to arousal responses. Collectively, these results demonstrated contrasting profiles: Hinoki aligned with calm energy and camphor with tense energy, providing the rationale for selecting these two odors for comparison in Experiment 2.

Experiment 2 confirmed general physiological relaxation, as SCL and HR decreased over time during rest. Planned comparisons revealed that HR immediately after odor introduction was lower under the Hinoki condition compared to no odor, indicating facilitated initial recovery of ANS activity, whereas camphor showed no such effect. This suggests that Hinoki, positioned as calm energy, may contribute to stabilization immediately after stress exposure. In contrast, SCL showed no condition differences, and neither HRV (HF, LF/HF) nor O_2_Hb showed significant effects. Prior studies have similarly reported HR reductions associated with inhalation of Hinoki essential oil [13], consistent with the present findings. For psychological indices, both TA and EA decreased across rest periods regardless of condition, but no between-condition differences were detected. Thus, quadrant differences appear to influence primarily the initial phase of ANS recovery, while effects on CNS indices remained inconclusive.

Taken together, these findings partially support hypothesis H2 proposed in the Introduction: the TA axis reflects tendencies in ANS responses, while the EA axis relates to CNS and vitality-related indices. Hinoki, located in the calm energy quadrant, was associated with HR reduction, whereas camphor, located in tense energy, did not differ from no odor. Psychological arousal showed tendencies consistent with quadrant positioning, but without conclusive evidence; for CNS indices, the directional hypothesis remains provisional. Mechanistically, contributions from monoterpenes such as 1,8-cineole and linalool are likely. Differences between wood flour and essential oils suggest additional roles of emission rates, substrate-derived naturalness, and higher-order cognitive factors such as expectation and association. The attenuation of odor intensity and impressions over short periods indicates the influence of olfactory adaptation and habituation. Future studies should incorporate GC–MS profiling, controlled concentration series or intermittent presentation, and experiments controlling for visual information and semantic associations.

The present findings highlight the potential of quadrant mapping for functional olfactory design. For short-term recovery immediately after stress, calm energy types such as Hinoki appear effective, whereas tense energy types such as camphor may be useful for maintaining or reactivating arousal. However, effectiveness varies with presentation mode (material vs. essential oil), concentration, and exposure duration, and olfactory adaptation may rapidly diminish perceived intensity. The presence of wooden materials themselves may reduce TA by enhancing perceived naturalness, indicating that combining diffused scents with material introduction could be a promising strategy.

This study also had limitations, including small sample size, restricted age range and gender balance, limited external validity of the presentation method, and difficulties in the intensity matching. Another limitation concerns the short duration of odor exposure. This design intentionally focused on the initial recovery phase following acute stress, as prolonged exposure may cause olfactory adaptation and reduce responsiveness. Future studies should compare different exposure durations to evaluate sustained effects and adaptation mechanisms over time. Analyses in Experiment 2 were based on baseline-differenced HR values at each time point; future work should employ linear mixed models incorporating random effects or intercept-based analyses of immediate responses. HRV and NIRS indices may also benefit from improved sensitivity through refined analytical methods and task designs.

Building on these limitations, further research should replicate quadrant effects with larger and more diverse samples, identify chemical profiles via GC–MS, and systematically control concentration series and intermittent presentation. In addition, validation under multisensory conditions that include visual and tactile cues will be essential to improve both the generalizability and the practical precision of olfactory design guidelines.

## 5. Conclusions

This study mapped a wide range of olfactory stimuli, including those derived from wood, on the two-dimensional axes of TA and EA, and examined whether quadrant differences influenced recovery following stress. This study provides a functional and reproducible method for treating olfaction as a designable element.

In Experiment 1, wood flours, wood essential oils, and other essential oils were positioned on the TA–EA plane under uniform conditions. Wood flours tended to occupy relatively low-EA positions, whereas corresponding essential oils tended to shift toward higher EA. These findings support hypothesis H1: differences in odor type, chemical composition, and presentation mode (wood flour vs. essential oil) systematically influence TA/EA coordinates of stimuli.

In Experiment 2, based on the results of Experiment 1, Hinoki (calm energy type: low TA, high EA) and camphor (tense energy type: high TA, high EA) were selected to test recovery processes during post-task rest. In addition to the overall relaxation observed during rest, HR immediately after odor introduction was lower in the Hinoki condition than in the no-odor condition, indicating facilitated initial recovery of ANS activity. By contrast, camphor showed no difference from no odor, and no clear condition effects were detected for SCL, HRV, or O_2_Hb. These results support hypothesis H2—that the TA axis corresponds to ANS tendencies and the EA axis to CNS and vitality-related indices—at least for the initial phase of ANS recovery.

For practical applications, the TA/EA map provides a useful guide for odor selection according to purpose. Calm energy types (e.g., Hinoki) appear suitable for short breaks and stabilization immediately after stress, whereas tense energy types (e.g., camphor) may be effective for maintaining or reactivating arousal. Furthermore, the presence of wooden materials may contribute to reduced EA via enhanced perceived naturalness, suggesting that combining material introduction with scent diffusion, optimizing concentration, intermittent presentation, and exposure duration, and accounting for olfactory adaptation are important for implementation.

Considering the limitations discussed in the Discussion section, future research should aim to replicate these findings with larger and more diverse samples and extend validation to multisensory conditions, thereby improving the generalizability and applicability of quadrant-based olfactory design.

In summary, olfactory design based on the TA–EA map offers a robust rationale for intentionally creating indoor environments that “reduce tension without excessively diminishing vitality.” The present findings demonstrate that quadrant differences yield practically meaningful effects on at least the initial recovery of ANS activity, underscoring the value of quadrant mapping as a design framework for indoor olfactory environments.

## Figures and Tables

**Table 1:** Principal component scores and arousal levels elicited by all odors.

		Principal Component Scores		Arousal Levels	
		1	2	TA	EA
Wood flours	Hinoki (0.5 g)	8.3	11.0	19.3	26.3
	Hinoki (0.15 g)	6.8	9.8	19.2	25.4
	Hiba	4.1	6.3	20.5	24.7
	Camphor	−3.5	9.1	24.3	27.8
	Sugi	9.7	6.8	17.8	21.8
	Japanese White Pine	6.9	6.9	19.8	23.7
Wood essential oils or chemical components	Sugi oil	4.8	6.9	19.4	26.4
	Hinoki oil	5.5	10.9	21.3	27.3
	α-Pinene	1.5	7.7	21.2	27.1
	Limonene	4.7	9.6	19.3	27.3
	1,8-Cineole	−8.4	9.0	25.0	28.5
	Linalool	4.3	9.3	18.8	25.9
	α-Terpineol	0.6	5.2	20.8	25.3
Other essential oils	Bergamot	3.3	9.6	20.8	27.5
	Lavender	0.1	7.2	22.8	26.2
	Lemon	3.0	10.2	20.0	27.6
	Peppermint	−6.9	11.0	25.0	29.6
	Ylang ylang	0.3	4.3	23.2	23.6
	Grapefruit	3.0	11.3	21.3	26.8

**Table 2:** Correlations between subjective impressions and JUMACL scores (N = 19,**: *p* < 0.01, *: *p* < 0.05).

		Impressions									Arousal Levels	
	1	2	3	4	5	6	7	8	9	10	11	12
1. Intensity		−0.87 **	0.54 *	−0.86 **	0.40	−0.89 **	−0.57 *	−0.57 *	−0.65 **	0.03	0.95 **	0.60 **
2. “stinging -mild”			−0.63 **	0.87 **	−0.36	0.90 **	0.71 **	0.67 **	0.74 **	0.09	−0.87 **	−0.61 **
3. “misty -clear”				−0.42	0.89 **	−0.63 **	−0.10	0.01	−0.31	0.54 *	0.40	0.90 **
4. “fidgety -relaxed”					−0.23	0.86 **	0.76 **	0.74 **	0.78 **	0.27	−0.90	−0.47 *
5. “depressed -refreshed”						−0.38	0.21	0.31	−0.08	0.68 **	0.22	0.79 **
6. “irritated -calm”							0.73 **	0.63 **	0.72 **	0.03	−0.86 **	−0.68 **
7. “bad -good”								0.94 **	0.76 **	0.57 *	−0.70 **	−0.19
8. “dislike -like”									0.72 **	0.61 **	−0.72 **	−0.13
9. “artificial -natural”										0.49 *	−0.72 **	−0.41
10. “unfamiliar -familiar”											−0.18	0.38
11. TA												0.49 *
12. EA												

## Data Availability

The data that support the study findings are available from the corresponding author upon reasonable request.

## Abbreviations

TA: Tense Arousal, EA: Energetic arousal, ART: Attention Restoration Theory, ANS: Autonomic Nervous System, CNS: Central Nervous System, JUMACL: the Japanese version of the UWIST Mood Adjective Checklist, PCA: Principal Component Analysis, SCL: Skin conductance Level, HR: Heart Rate, HRV: Heart Rate Variability, O_2_;Hb: Oxygenated Hemoglobin concentration, LF: Low Frequency, HF: High Frequency, NIRS: Near-Infrared Spectroscopy.
